# Fluoxetine for Autistic Behaviors (FAB trial): study protocol for a randomized controlled trial in children and adolescents with autism

**DOI:** 10.1186/1745-6215-15-230

**Published:** 2014-06-16

**Authors:** Anissa Mouti, Dinah Reddihough, Catherine Marraffa, Philip Hazell, John Wray, Katherine Lee, Michael Kohn

**Affiliations:** 1 Centre for Research into Adolescent’s Health (CRASH), Sydney Children’s Hospital Network, Westmead Sydney Medical School, The University of Sydney, Hawkesbury Road, 2145 Sydney, Australia; 2 Discipline of Psychiatry, Sydney Medical School, The University of Sydney, Hospital Rd, Concord West, 2138 Sydney, Australia; 3 Department of Pediatrics, University of Melbourne, Flemington Road, 3052 Parkville, Australia; 4 Murdoch Children’s Research Institute, Flemington Road, 3052 Parkville, Australia; 5 State Child Development Centre, Rheola Street, 6872 West Perth, Australia

**Keywords:** Autism Spectrum Disorder (ASD), Autism, Serotonin Reuptake Inhibitors (SSRIs), Fluoxetine, Repetitive and Restricted Behaviors, Randomized Controlled Trial (RCT), Drug Therapy, Children, Adolescents, Safety and Efficacy

## Abstract

**Background:**

Serotonin reuptake inhibitors (SSRIs) are commonly prescribed off-label for children with autism. To date, clinical trials examining the use of SSRIs in autism have been limited by small sample sizes and inconclusive results. The efficacy and safety of SSRIs for moderating autistic behaviors is yet to be adequately examined to provide evidence to support current clinical practice. The aim of the Fluoxetine for Autistic Behaviors (FAB) study is to determine the efficacy and safety of low dose fluoxetine compared with placebo, for reducing the frequency and severity of repetitive stereotypic behaviors in children and adolescents with an autism spectrum disorder (ASD). The relationship between the effectiveness of fluoxetine treatment and serotonin transporter genotype will also be explored.

**Methods/Design:**

The FAB study is a multicenter, double-blinded, randomized controlled trial, funded by the Australian Government’s National Health and Medical Research Council (NHMRC) grant. Participants will be aged between 7.5 and 17 years with a confirmed diagnosis of ASD. Eligible participants will be randomized to either placebo or fluoxetine for a 16-week period. Medication will be titrated over the first four weeks. Reponses to medication will be monitored fortnightly using the Clinical Global Impressions Scale (CGI). The primary outcome measure is the Children’s Yale-Brown Obsessive Compulsive Scale-Modified for Pervasive Developmental Disorders (CYBOCS-PDD), administered at baseline and 16 weeks. Secondary outcome measures include the Aberrant Behaviour Scale (ABC), the Spence Children’s Anxiety Scale Parent Report (SCAS-P), and the Repetitive Behaviors Scale (RBS-R), measured at baseline and 16 weeks. Participants will be invited to undergo genetic testing for *SLC6A4* allele variants using a cheek swab. Continuous outcomes, including the primary outcome will be compared between the active and placebo groups using unadjusted linear regression. Binary outcomes will be compared using unadjusted logistic regression.

**Discussion:**

The FAB study is a large clinical trial to specifically investigate the efficacy of low dose fluoxetine for restricted, repetitive, and stereotyped behaviors in ASD. The outcomes of this study will contribute to evidence-based interventions used in clinical practice to assist children with ASD.

**Trial registration:**

Australian and New Zealand Clinical Trials Registry ACTRN12608000173392; registered on 9 April, 2008.

## Background

In 2006, the Autism Council of Australia reported that 1 in 160 children in Australia are diagnosed with an autism spectrum disorder (ASD) [1]. ASDs are neurodevelopmental disorders characterized by impairments in social communication and interaction and a pattern of restricted, repetitive, and stereotyped interests and behaviors. Associated symptoms include anxiety, irritability, aggression, and self-injury. The fifth and most recent edition of the Diagnostic and Statistical Manual of Mental Disorders (DSM-5) aggregates the former Diagnostic and Statistical Manual of Mental Disorders Text Revision Fourth Edition (DSM-IV-TR) conditions of autism disorder, Asperger’s disorder and pervasive developmental disorder-not otherwise specified (PDD-NOS) into one ASD in recognition of the continuum of severity seen with the condition [2].

Repetitive behaviors constitute a core feature of ASD and include repetitive motor phenomena (such as stereotypies), repetitive speech and language (such as echolalia), restricted and repetitive play, a narrow range of interests, overwhelming preoccupations, obsessions, routines and rituals, and resistance to change [3]. These behaviors may be associated with high levels of anxiety and self-injury. Such behaviors typically result in significant functional impairment for the affected individual and impede their quality of life, as well as creating a significant burden for their families. Follow-up studies have found that only between 3 and 10% of people with autism are able to live independently as adults [4-6]

Given the heterogeneous nature of difficulties observed for an individual on the autism spectrum, it is often difficult to discern which individuals will benefit from the array of available interventions. The mainstay of interventions for individuals with autism is individualized strategies to facilitate communication, social interaction, and behaviour management. However, pharmacotherapy can also play a role in the management of targeted symptoms with the aim of reducing behaviors that are interfering, making individuals more responsive to educational and behavioral interventions. Approximately 21 to 32% of children with autism are prescribed an antidepressant medication, which is most often a selective serotonin reuptake inhibitor (SSRI) [7-9]. Despite their common use, regulatory bodies are yet to approve the use of SSRI medication for autism.

In addition, there is evidence that serotonin plays a contributory role in the pathophysiology of autism, with converging support from genetic, biologic, and neuroimaging studies indicating that individuals with ASD have higher serotonin levels than those without ASD [10]. Depletion of the serotonin precursor tryptophan has also been shown to induce a worsening of autistic symptoms (such as hand flapping and circumscribed interests) in adults with autism [11]. A correlation between the SSRI, fluvoxamine, and the ‘L’ variant genotype of the serotonin transporter gene (*SLC6A4*) was found in a study of 18 children with autism, supporting a link between serotonin and ASD and providing preliminary support for the effectiveness of SSRIs as an alternative treatment for ASD [12]. Evidence for the clinical benefit of SSRIs in treating patients with autism is, however, equivocal. A Cochrane review [4] identified nine randomized placebo-controlled trials (320 people) of SSRIs for children and adults with autism. The findings of the review were inconclusive. The limitations of the included studies were small sample sizes and variability in the primary and secondary outcome measures. The review identified a need for adequately powered clinical trials of SSRIs that use a standardized battery of outcome measures. It has been suggested that SSRIs can cause dose-related harm to children with autism such as insomnia, motor hyperactivity, agitation, and aggression [13], a finding that also warrants further investigation.

The aim of the present study is to contribute to the knowledge base concerning the effectiveness and tolerability of SSRIs in children and adolescents with autism through the conduct of a large, multicenter randomized controlled trial of low dose fluoxetine versus placebo. The study will focus on fluoxetine because it is one of the most commonly prescribed SSRI agents in children with autism.

### Study aims

The aims of the FAB study are:(1) to determine the efficacy and safety of low dose fluoxetine compared to placebo for reducing the frequency and severity of restricted, repetitive, and stereotypic behaviors in children and adolescents with an ASD; and (2) to explore the relationship between the effectiveness of low dose fluoxetine and the serotonin transporter (*SLC6A4*) genotype.

## Methods/Design

### Study design

The FAB study is a multicenter randomized, double-blinded, placebo-controlled study with a parallel-group design. Treatment for both active and control groups will be of 16 weeks duration. After 16 weeks, participants will be tapered off the study medication over a four-week period and followed up on for a further two weeks. The study is funded by the National Health and Medical Research Council of Australia (NHMRC project grant number: 607332). The study was registered on 9 April 2008 with the Australian and New Zealand Clinical Trials Registry (ACTRN12608000173392). The study was approved by Human Research Ethics Committees (HRECs) at each site: The Royal Children’s Hospital Human Research Ethics Committee (EC00238) for the site in Victoria, the Sydney West Area Health Service Human Research Ethics Committee, Westmead Campus (EC00152) for the site in New South Wales and the Princess Margaret Hospital for Children Ethics Committee (EC00268) for the site in Western Australia.

### Participants

Participants will need to meet eligibility criteria requirements in order to participate in the trial. Eligibility criteria for the study includes a confirmed diagnosis of ASD, based on the Autism Diagnostic Interview-Revised (ADI-R) and DSM-IV-TR [14,15], and aged between 7.5 and 17 years.

The presence of any exclusion criteria will preclude the participant from enrolling into the FAB study. Details of all participants deemed ineligible will be recorded. Exclusion criteria for the trial include a known DSM-IV diagnosis of Rett’s Disorder, Childhood Disintegrative Disorder, Schizophrenia or Major Depression. Patients currently prescribed or who have received in the previous six weeks prior to the trial other psychotropic medications, including other SSRI’s, typical and atypical anti- psychotics, mood stabilisers, and anxiolytic medication, monoamine oxidase inhibitor (MAOI) or pimozide, antidepressants, St John's Wort and/or atomoxetine will be excluded from the trial. Patients that have concomitant administration of drugs that interact with the metabolism of fluoxetine (such as phenytoin or carbamazepine) or drugs contraindicated for use with fluoxetine (such as monoamine oxidase inhibitors and pimozide) will be ineligible to take part in the study. Patients who have co-morbid significant medical conditions (such as unstable seizure disorder, cardiac disease, liver failure or renal failure) will not be eligible for the study. Female patients who are pregnant will not be allowed to participate in the study.

Stimulant medications were on the exclusion list of drugs for the Western Australian site (see site recruitment) only.

### Site recruitment

The FAB trial will be conducted across three Australian sites: The Royal Children’s Hospital (Victoria), The Children’s Hospital at Westmead (New South Wales), and the State Child Development Centre (Western Australia).

Clinical sites were based on the likelihood of meeting recruitment goals and executing the protocol. All three centers care for similar numbers of children and adolescents with ASD. As such, it is thought that they will contribute equally to recruitment. Participants will be recruited through the following referral pathways: pediatricians, child and adolescent psychiatrists, psychologists, and general practitioners. Professionals who treat children and/or adolescents with autism at or around the above institutes will be sent a letter explaining the study, along with a study synopsis and copies of the study brochure for parents and/or guardians. Clinical trial coordinators will work together with clinicians who have been approached and are assisting with the study to ensure compliance with protocol guidelines and safety monitoring.

### Enrollment and randomization

After obtaining written consent from participants and/or their caregivers as per protocol guidelines, eligible participants will be randomized to the active or control group in a 1:1 ratio according to a computer-generated randomization schedule prepared by the Clinical Epidemiology and Biostatistics Unit (CEBU) at the Royal Children’s Hospital. Block randomization will be used with variable block sizes, with randomization stratified by site, age (7.5 to under 12 years and 12 to 17 years) and IQ (IQ ≥70: average or borderline range and IQ <70: intellectual disability). Thus there are a total of 12 strata (4 per site). The randomization schedule for each site will be given to the clinical trials pharmacist appointed at each site, who will then be responsible for arranging a sequential stock of trial medication for each stratum, labelled with only the study number, strata, and instructions for use. This schedule will remain confidential throughout the trial. The independent statistician from CEBU will also keep a copy of the master randomization schedule to check for any discrepancies. As each participant is enrolled in the study they will be allocated the next ID code within the correct stratum.

### Interventions

The active and placebo medication will be produced by Richard Stenlake Chemists (Bondi Junction, Australia). Participants randomized to the active group will receive fluoxetine syrup (2 mg/ml), which contains fluoxetine hydrochloride dissolved in a methocel base (containing glycerine, polysorbate 80, sodium saccharin, citric acid, methocel E4M, sodium benzoate and water). Participants in the control group will receive placebo syrup which will be the methocel base only. Both the active and placebo medication are transparent red syrups with raspberry flavouring. The packaging for both the active and placebo syrups will be identical with containers labelled only with Study Syrup and the study ID number. The medication will be packed into 250 ml bottles.

### Dispensing and blinding

Medication will be dispensed by clinical trials pharmacists at each site according to randomization allocation. Parents and/or guardians, participants, and study investigators will remain blinded to the treatment allocation until the completion of the study. The randomization schedule will be known only to CEBU and the clinical trials pharmacist at each site.

### Assessments and measures

#### Screening assessments

As part of the screening assessment, standardized assessment tools will be administered for ASD and cognitive functioning. Specifically, to assess for ASD, the ADI-R [16] which is a standardized diagnostic interview will be used to obtain information from parents and/or caregivers about their child’s developmental history and current level of functioning. The interview can take up to three hours to complete. Information is scored onto an algorithm. There are two algorithms, the current behaviour algorithms to ascertain a child’s current functioning, and the diagnostic algorithms for a formal assessment to produce an ADI-R diagnosis. For the purposes of the study, only the diagnostic algorithms will be used to ensure participants meet criteria for ASD.

When assessing cognitive functioning, participants who do not have previously documented cognitive assessments will complete the Wechsler Intelligence Scale for Children Fourth Edition (WISC-IV), Wechsler Adult Intelligence Scale Fourth Edition (WAIS-IV), or Wechsler Nonverbal Scale of Ability (WSN) according to their age and level of expressive communication as determined by the treating clinician. The WISC-IV [17]: is the most commonly used clinical tool for assessing the general thinking and reasoning skills of children aged between 6 and 16-years-old. The WISC-IV consists of four index scores: the Verbal Comprehension Index (VCI), Perceptual Reasoning Index (PRI), Working Memory Index (WMI), and Processing Speed Index (PSI), each containing several subtests. The Full Scale IQ Score (FSIQ) is a composite score comprised of these four index scores and serves to provide a global measure of general intellectual ability.The WAIS*-*IV [18] is the most commonly used clinical tool for assessing the general thinking and reasoning skills of adults aged 16 to 90-years-old. The WAIS-IV consists of four index scores: the VCI, PRI, WMI, and PSI, each containing several subtests. The FSIQ is a composite score comprised of these four index scores and serves to provide a global measure of general intellectual ability The WNS [19] is the clinical tool used for assessing the general thinking and reasoning skills of individuals aged 4 years to 21 years 11 months, who have limited language skills, language disorders, deafness, or diverse linguistic groups. The FSIQ is a composite score comprised of subtest scores. The difference between this tool and the above Wechsler measures is that it eliminates the need for verbal communication and is therefore an optimal tool for nonverbal or minimally verbal child populations.

#### Baseline and follow-up measures

The primary outcome measure (measured at baseline and 16 weeks) for the trial will be the Children*’*s Yale*-*Brown Obsessive Compulsion Scale*-*Modified for Pervasive Developmental Disorders (CYBOCS*-*PDD). The CYBOCS includes a detailed symptom checklist of possible obsessions and compulsions which are then rated across five severity items (time spent on obsessions, interference, distress, resistance, and degree of control) [20]. The CYBOCS has been modified for use in children with a pervasive developmental disorder (PPD). Both the CYBOCS-PDD and YBOCS (adult version) have been widely used in clinical drug trials for autism and have been demonstrated to be sensitive to change [13,21]. The CYBOCS-PDD score was chosen as the primary outcome measure for this trial, in line with previous trials in this field.

The secondary outcome measures (measured at baseline and 16 weeks) will be the Repetitive Behaviors Scale*-*Revised (RBS*-*R). This revised version of the scale captures the breadth of repetitive behaviors in autism. The RBS-R consists of six subscales: stereotypic behaviors, self-injurious behaviors, compulsive behaviors, ritualistic behaviors, sameness behaviors, and restricted behaviors. Recently, the internal validity and interrater reliability of the RBS-R was validated in both children and adults with autism [22]. The Spence Children*’*s Anxiety Scale (SCAS) will be another secondary outcome measure used The SCAS scale consists of a child and parent version. The scale contains six subscales (panic/agoraphobia, social anxiety, separation anxiety, generalized anxiety, obsessions/compulsions, and fear of physical injury) and provides a total score. The validity and reliability of the SCAS (child and parent versions) has been established for both anxiety-disordered and normal control populations. The parent version was used for the purpose of this study [23-25]. The Aberrant Behaviour Checklist (ABC) - Community Version will be a secondary outcome measure to assess maladaptive behaviors in individuals with developmental disability or intellectual impairment. The ABC items are grouped into five subscales: irritability/agitation, lethargy/social withdrawal, stereotypic behaviour, hyperactivity/non-compliance, and inappropriate speech. The ABC has been widely used in clinical drug trials in autism. The factor structure of the ABC - community version has been found to be robust in an ASD sample of 275 individuals aged between 3 and 21 years [26]. The Clinical Global Impressions Scale (CGI) is a widely used scale that assesses treatment response in psychiatric conditions and will be used as a secondary outcome measure. It is a three-item scale: severity of illness (rated on a 7-point scale), global improvement (rated on a 7-point scale), and efficacy index (rated on a 4-point scale) [27]. The CGI has been widely used in clinical drug trials in autism, and has been shown to be sensitive to change [12,13,27].

### Procedures

#### Study regimens

**Figure 1:**
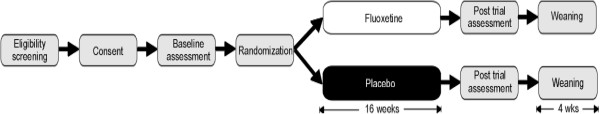
FAB consort flow diagram.

Figure 1 illustrates the schema for the FAB study. Potentially eligible participants will complete pretrial assessment measures prior to commencing medication. After pretrial assessments, eligible participants will be randomized to either an active group or a control group. Fluoxetine or placebo syrup will be administered orally, once daily, in the morning. The study medication (fluoxetine or placebo) doses will be based on the participant’s weight. Study medication will be commenced at 4 or 6 mg/day for the first week (4 mg if <40 Kg, 6 mg if ≥40 Kg). The medication will then be titrated up at weekly intervals, over the subsequent three weeks, using a flexible titration schedule (See Table 1 and Table 2). The maximum dose used will be 30 mg/day for participants greater than or equal to 40 kg, and 20 mg/day for participants who are less than 40 kg. No further dose increases will occur after week four of the trial. The dose may be decreased (or the medication ceased) at any time during the trial should significant side effects occur. At 16 weeks, a follow-up visit will be conducted in which post-assessment measures will be completed. There will then be a four week taper. Routine dosage adjustments and side effect monitoring will be performed by phone interviews on a weekly basis for the first four weeks and then fortnightly.

**Table 1:** Summary of fluoxetine dosing schedule for participants <40 Kg

**Week of trial**	**Dose (per day)**
1	4 mg
2	8 mg
3	14 mg
4	20 mg
5-16	Maintain effective dose
17-20	Wean off medication (at weekly intervals)

**Table 2:** **Summary of fluoxetine dosing schedule for participants** ≥**40 Kg**

**Week of trial**	**Dose (per day)**
1	8 mg
2	14 mg
3	22 mg
4	30 mg
5-16	Maintain effective dose
17-20	Wean off medication (at weekly intervals)

### Participant study visits

#### Baseline, medical and genetic testing

Participants in the study are required to meet the clinician on three occasions, two appointments for baseline assessment and one appointment to study sites for the post-trial assessment. During the first visit the study doctor will take a detailed medical history and conduct a physical examination. For participants who do not have previously documented cognitive testing, the WISC-IV, WNV, or another appropriate measure will be administered by a psychologist during the visit. In addition, consent will be obtained from the parents and a cheek-brush sample will be collected for genetic analysis of the serotonin transporter gene (*SLC6A4*). The second visit will involve a confirmation of the participant’s ASD diagnosis by a trained psychologist using the ADI-R and DSM-IV-TR criteria.

During either of these baseline visits, baseline ratings will be obtained for the CYBOCS-PDD, RBS-R, SCAS-parent version, and the ABC-community version. Baseline ratings for the CGI-R will be obtained by the study doctor, psychologist, and/or research assistant. In addition, participant’s levels of anxiety, anger/aggression, sadness/depression, excitability/overexcited, and ADHD were rated on a scale of 0 to 2 by the study doctor, psychologist, and/or research assistant. Following the second visit, the participant is randomized and study medication commencement is arranged.

#### Post-treatment assessments

At the completion of the 16-week treatment period, the participant and their parent(s) will be asked to return to the hospital for another study visit. During this visit ratings will be obtained for the CYBOCS-PDD, RBS-R, SCAS-parent version, ABC-community version, and the CGI. The participant's levels of anxiety, anger/aggression, sadness/depression, excitability/overexcited, and AD/HD were rated on a scale of 0 to 2 by the study doctor, psychologist, and/or research assistant. The participant is then weaned off the study medication over a four-week period and monitored weekly by either the research assistant or the study doctor.

#### Treatment delivery

The dosage schedule for the study medication, summarized in Tables 1 and 2, is based on previous research that highlights the importance of using lower doses and titrating doses slowly when treating children and adolescents with autism [13,28,29]. Dosages will be monitored using medication diaries provided to participants and their parents by the treating clinician. Participants will be given three medication diaries that outline the amount of medication to be taken at given periods: ‘Diary 1’ for weeks 1 to 4, in which titration levels are increased; ‘Diary 2’ for weeks 5 to 16, in which medication dosages are consistent and maintained; and ‘Diary 3’ for weeks 17 to 22, in which medication is tapered. Parents are required to return each booklet at the required interval to monitor medication compliance.

### Monitoring, governance and withdrawal

#### Quality assurance and training

Trial psychologists who will carry out the assessments will have the required experience and training to administer psychometric testing and will undertake training to obtain a research accreditation for the administration of the ADI-R if they do not already have this training. Trial doctors (pediatricians or psychiatry registrars) will be supervised by a chief investigator (pediatrician or psychiatrist) as well as a trial coordinator at each site to ensure protocol compliance.

#### Adverse events and safety monitoring

A medical history and physical examination will be conducted prior to the commencement of the study. This will assist in the interpretation of adverse events if they occur during the study. Monitoring for adverse events will occur by phone consultations on a weekly basis during tapering up and weaning down of the study medication. During weeks 4 to 15, participants will be monitored on a fortnightly basis. All adverse events reported by the participant and/or caregiver will be thoroughly evaluated and appropriate measures taken as per trial protocol guidelines. The nature of each adverse event, time of onset, duration, severity, and relationship to treatment will be established and recorded. Details of any changes to the dosage schedule or any corrective treatment will be recorded. All serious adverse events will be reported to the relevant Human Research Ethics Committee and Adverse Drug Reaction Committee, and well as the study Independent Data Safety Monitoring Committee (DSMC). The clinical trials coordinator will also be available to assist if necessary. Participants are able to contact coordinators at any given time should they be concerned about potential adverse side effects.

#### Patient completion and withdrawal

Subjects will be considered to have finished the study upon completion of 16 weeks of the study medication. Premature termination of the study drug will be defined as completing less than 16 weeks of treatment. Even if the study drug is terminated, every effort will be made by the investigator to maintain data collection until study completion. A patient may withdraw (or be withdrawn) from treatment prematurely because of significant adverse events, or a patient or caregiver decision to discontinue the treatment.

### Data collection

#### Case report forms (CRF)

All study data will be captured on a CRF. The CRF will contain the patient’s study number, date of each consultation (including phone consultations), results of pre and post assessments, and adverse events, and include medications that participants may be taking that are not on the exclusion list.

#### Web-based database

An online database will be developed for the trial, with data entry occurring at each site. Original CRFs will be used when entering information into the computer database, by the study coordinator at each site.

#### Serotonin transporter gene

To determine a relationship between an individual’s serotonin transporter genotype and response to treatment with fluoxetine compared to placebo in children and adolescents with autism (Secondary Aim 2 of the study), genetic samples will be collected and sent from study sites and de-identified prior to processing at the Bruce Lefroy Centre, Murdoch Children’s Research Institute. Samples will be collected by a cheek brush and genotypes determined by polymerase chain reaction (PCR using Bio-Rad iCycler, California) and gel electrophoresis analysis.

#### Sample size

Previous data from 172 medication-free children with ASD showed a mean total score of 14.4, with a standard deviation (SD) of 3.86 on the CYBOCS-PDD [27]. Based on the study investigators’ clinical experience in the management of children with ASD, it was thought that a difference of 2 on the CYBOCS-PDD would represent a clinically important improvement in repetitive behaviors (approximately 0.5 of a standard deviation). In order to find an effect size of 0.5 (corresponding to a difference of 2 on the CYBOCS-PDD based on a standard deviation of 3.8) with 80% power and a two-sided alpha of 0.05 requires a sample size of 64 per treatment group. Allowing for a 15% attrition rate, we therefore plan to recruit 73 participants per treatment group for a total of 146 participants.

#### Statistical analysis

Analysis will be performed on an intention-to-treat basis, including all participants with outcomes data available.

#### Baseline analyses

Baseline characteristics will be presented separately for children in the active and placebo groups using means and standard deviations for continuous data (or medians and interquartile ranges for non-normal data) and proportions for categorical data.

#### Primary outcome

The primary outcome (total score on the CYBOCS-PDD at 16 weeks) will be summarized by mean and standard deviation (SD) treatment group, with the comparison between active and placebo presented as the difference in means and the 95% confidence interval (CI), obtained using an unadjusted linear regression model. As a sensitivity analysis, the regression models will also be adjusted for pre-trial CYBOCS-PDD score, age, and IQ at baseline.

#### Secondary outcomes

Secondary continuous outcomes will be compared between the active and control groups using unadjusted linear regression models, and binary outcomes using unadjusted logistic regression. As a sensitivity analysis regression models will also be fitted to the secondary outcomes adjusted for pretrial scores on the questionnaire of interest, age, and IQ at baseline (as used in the randomization).

## Discussion

Currently, SSRIs are prescribed “off-label” for the treatment of children with autism.To date, the results of clinical trials of SSRIs have been mixed and inconclusive. Importantly, studies have not proven that SSRIs are effective in treating autism. Despite precautions of prescribing SSRIs to children, the off-label use of fluoxetine among clinicians is becoming widespread and acceptable. An adequately powered well-designed randomized control trial of SSRIs is needed. The FAB study is, to our knowledge, the largest clinical trial to specifically investigate the efficacy of low dose fluoxetine for restricted, repetitive, and stereotyped behaviors in ASD. The outcomes of this study will contribute to evidence-based interventions used in clinical practice to assist children with ASD.

## Trial status

The first participant enrolled in the study was 15 November, 2010. At the time of this manuscript submission, participants were still being recruited.

## Abbreviations

ABC: Aberrant Behaviour Scale; ADI-R: Autism Diagnostic Interview; ASD: Autism Spectrum Disorder; CEBU: Clinical Epidemiology and Biostatistics Unit at the Royal Children’s Hospital; CGI: Clinical Global Impressions Scale; CRF: Case Report Forms; CYBOCS-PDD: Children’s Yale-Brown Obsessive Compulsive Scale; DSM-IV-R: Diagnostic and Statistical Manual of Mental Disorders Fourth Edition Text Revision; FAB: Fluoxetine for Autistic Behaviors; MCRI: Murdoch Children’s Research Institute; PCR: Polymerase Chain Reaction; RBS-R: Repetitive Behaviors Scale; SCAS-parent version: Spence Children’s Anxiety Scale parent version; SLC6A4: Serotonin Transporter Gene; SSRIs: Selective Serotonin Reuptake Inhibitors; WAIS-IV: Wechsler Adult Intelligence Scale; WISC-IV: Wechsler Intelligence Scale for Children, 4th edition; WNV: Wechsler Non-Verbal Scale of Ability.

## Competing interests

PH and MK have received payment from Eli Lilly (the manufacturer of fluoxetine) for participation in consultancies, advisory boards, speaker’s bureau, and the conduct of clinical trials.

## Authors’ contributions

DR, MK, CM, and JW assisted in the conception and design of the study and are involved in recruitment and the ongoing conduct of the study. KL contributed to the statistical design of the study. PH participated in the design and coordination of the study and helped to draft this manuscript. AM drafted this manuscript and is involved in recruitment of the study. All authors read and approved the final manuscript.
